# Multi-scale CFD simulation of hydrodynamics and cracking reactions in fixed fluidized bed reactors

**DOI:** 10.1007/s13203-015-0130-1

**Published:** 2015-08-15

**Authors:** Jin H. Zhang, Zhen B. Wang, Hui Zhao, Yuan Y. Tian, Hong H. Shan, Chao H. Yang

**Affiliations:** State Key Laboratory of Heavy Oil Processing, China University of Petroleum, Qingdao, 266580 China

**Keywords:** Multi-scale structure, Simulation, Catalytic cracking, Reaction kinetics, Fixed fluidized bed reactor

## Abstract

Fixed fluidized bed reactor is widely used to evaluate the crackability of heavy oils and the activity of catalysts. To understand the hydrodynamics, reaction kinetics and thermodynamics in conventional and modified fixed fluidized bed reactors, the computational fluid dynamics method, energy-minimization multi-scale-based two-fluid model coupled with a six-lump kinetic model was used to investigate the gas–solid flow and cracking reactions. The gas mixing and particle volume fraction distributions, as well as product yields in the conventional and modified fixed fluidized bed reactors were analyzed. The residence time distribution model was utilized to obtain the parameters indicating the back-mixing degree, such as mean residence time and dimensionless variance of the gas. The results showed that the simulated product distribution is in reasonable agreement with the experimental data; the modified fixed fluidized bed reactor is closer to the ideal plug flow reactor, which can efficiently enhance the gas–solid mixing, reduce the gas back-mixing degree, and hence improve the reaction performance.

## Introduction

Fixed fluidized bed reactor is a kind of fluidized bed that has no external circulating particles. It usually has only one reactor, by setting a simple filter or cyclone to trap the particles. Fixed fluidized bed reactor has many advantages, such as low cost, high thermal efficiency, isothermal bed temperature and low operation and maintenance cost [1]. Therefore, the fixed fluidized bed reactor is widely used in laboratory studies for operating parameter optimization and catalyst evaluation [2, 3], as well as developing kinetic models [4, 5], especially for fluid catalytic cracking (FCC) process. However, little work on studying the gas–solid flow behavior in fixed fluid beds has been reported in the literature.

In a chemical reactor, on the one hand, reactions can significantly influence the internal gas–solid flow behavior, especially the existence of molecule numbers sharply increased reactions, such as in the heavy oil catalytic cracking process; on the other hand, gas–solid mixing behavior plays a remarkable role in determining the conversion and selectivity of chemical reactions.

The performance of a fixed fluidized bed reactor strongly depends on the interactions between oil and catalyst flows, but most heavy oil catalytic cracking reaction models only consider cracking kinetics such as five-lump kinetic model [4, 5], six-lump kinetic model [6] and seven-lump kinetic model [7]. For the time-resolved reaction process, besides gas–solid contact, gas residence time distribution is also very important to the product distribution; however few researchers have followed with interest.

With the improvement of computer power and numerical algorithms, computational fluid dynamics (CFD) has become a useful tool for studying the hydrodynamics in complex multiphase systems. Because of the low computational expense, the two-fluid model is widely used to describe the gas–solid fluidized bed on the basis of the assumptions [8] that gas and solid are treated as continuous and interpenetrating mediums. However, this model does not consider the effects of mesoscale structures, such as bubble and cluster, which will lead to a qualitatively incorrect conclusion [9, 10]. Therefore, the energy-minimization multi-scale (EMMS) model was developed, which modified the drag force by introducing a heterogeneous index to reflect the effect of mesoscale structures [11, 12] and has proven to be effective in simulating the high-density riser reactor [13–15].

In this work, the EMMS-based two-fluid model coupled with a six-lump kinetic model was used to investigate the gas–solid flow, heat transfer, mass transfer and reaction processes in conventional and modified fixed fluidized bed reactors. Gas residence time distribution, catalyst distribution and product distribution were analyzed to compare the two reactors.

## EMMS-based two-fluid model and six-lump kinetic model

### EMMS-based two-fluid model

In this paper, the EMMS-based two-fluid model was used to describe the mixing behavior in laboratory-scale fixed fluidized bed reactor. Numerical simulations are based on the commercial software, FLUENT^®^6.3.26. The basic equations given below represent the conversion of mass, momentum and energy for the gas and solid phases (Fluent User’s guide). The EMMS drag model, which had been described in detail elsewhere [11, 12], was incorporated into FLUENT through a user-defined function (UDF).

Continuity equations are as follows:

Gas phase:

1 $$\frac{\partial }{\partial t}(\varepsilon_{\text{g}} \rho_{\text{g}} ) + \nabla (\varepsilon_{\text{g}} \rho_{\text{g}} \vec{u}_{\text{g}} ) = 0.$$

Solid phase:

2 $$\frac{\partial }{\partial t}(\varepsilon_{\text{s}} \rho_{\text{s}} ) + \nabla (\varepsilon_{\text{s}} \rho_{\text{s}} \vec{u}_{\text{s}} ) = 0.$$

Momentum equations are as follows:

Gas phase:

3 $$\frac{\partial }{\partial t}(\varepsilon_{\text{g}} \rho_{\text{g}} \vec{u}_{\text{g}} ) + \nabla (\varepsilon_{\text{g}} \rho_{\text{g}} \vec{u}_{\text{g}} \vec{u}_{\text{g}} ) = - \varepsilon_{\text{g}} \nabla p_{\text{g}} + \nabla \overline{\overline{\tau }}_{\text{g}} + \varepsilon_{\text{g}} \rho_{\text{g}} \vec{g} + \beta (\vec{u}_{\text{s}} - \vec{u}_{\text{g}} ).$$

Solid phase:

4 $$\frac{\partial }{\partial t}(\varepsilon_{\text{s}} \rho_{\text{s}} \vec{u}_{\text{s}} ) + \nabla (\varepsilon_{\text{s}} \rho_{\text{s}} \vec{u}_{\text{s}} \vec{u}_{\text{s}} ) = - \varepsilon_{\text{s}} \nabla p_{\text{s}} + \nabla \overline{\overline{\tau }}_{\text{s}} + \varepsilon_{\text{s}} \rho_{\text{s}} \vec{g} + \beta (\vec{u}_{\text{g}} - \vec{u}_{\text{s}} ).$$

Stress of gas phase:

5 $$\overline{\overline{\tau }}_{\text{g}} = \mu_{\text{g}} \left\{ {[\nabla \vec{u}_{\text{g}} + (\nabla \vec{u}_{\text{g}} )^{\text{T}} ] - \frac{2}{3}(\nabla \vec{u}_{\text{g}} )\overline{\overline{I}} } \right\}$$

Stress of solid phase:

6 $$\overline{\overline{\tau }}_{s} = [ - P_{\text{s}} + \lambda_{\text{s}} (\nabla \vec{u}_{\text{s}} )]\overline{\overline{I}} + \mu_{\text{s}} \left\{ {[\nabla \vec{u}_{\text{s}} + (\nabla \vec{u}_{\text{s}} )^{\text{T}} ] - \frac{2}{3}(\nabla \vec{u}_{\text{s}} )\overline{\overline{I}} } \right\}.$$

Solid phase pressure:

7 $$P_{\text{s}} = \rho_{\text{s}} \varTheta_{\text{s}} + 2\rho_{\text{s}} \varTheta_{\text{s}} (1 + e)\varepsilon_{\text{s}} g_{0} .$$

Solid phase shear viscosity:

8 $$\mu_{\text{s}} = \mu_{\text{s,col}} + \mu_{\text{s,kin}} + \mu_{\text{s,fr}} ,$$

9 $$\mu_{\text{s,col}} = \frac{4}{5}\varepsilon_{\text{s}} \rho_{\text{s}} d_{\text{p}} g_{0} (1 + e)\sqrt {\frac{{\varTheta_{\text{s}} }}{\pi }} ,$$

10 $$\mu_{\text{s,skin}} = \frac{{10\rho_{\text{s}} d_{\text{p}} \sqrt {\varTheta_{\text{s}} \pi } }}{{96\varepsilon_{s} g_{0} (1 + e)}}\left[ {1 + \frac{4}{5}\varepsilon_{\text{s}} g_{0} (1 + e)} \right]^{2} ,$$

11 $$\mu_{\text{s,fr}} = 0.$$

Solid phase bulk viscosity:

12 $$\lambda_{\text{s}} = \frac{4}{3}\varepsilon_{\text{s}} \rho_{\text{s}} d_{\text{s}} g_{0} (1 + e)\sqrt {\frac{{\varTheta_{\text{s}} }}{\pi }} .$$

Radial distribution function:

13 $$g_{0} = \left[ {1 - \left( {\frac{{\varepsilon_{\text{s}} }}{{\varepsilon_{{{\text{s}},\hbox{max} }} }}} \right)^{1/3} } \right]^{ - 1} .$$

The granular temperature equation is as follows:

14 $$\frac{3}{2}\left[ {\frac{\partial }{\partial t}(\rho_{\text{s}} \varepsilon_{\text{s}} \varTheta_{\text{s}} ) + \nabla \cdot (\rho_{\text{s}} \varepsilon_{\text{s}} V_{\text{s}} \varTheta_{\text{s}} )} \right] = ( - P_{\text{s}} I + \tau_{\text{s}} ) \cdot \nabla V_{\text{s}} - \nabla \cdot (k_{{\varTheta_{\text{s}} }} \nabla \varTheta_{\text{s}} ) - \gamma \varTheta_{\text{s}} + \phi_{\text{gs}} .$$

15 $$\phi_{\text{gs}} = - 3\beta \varTheta_{\text{s}}.$$

Diffusion coefficient:

16 $$k_{{\varTheta_{\text{s}} }} = \frac{{150\rho_{\text{s}} d_{\text{s}} \sqrt {\varTheta_{\text{s}} \pi } }}{{384g_{0} (1 + e)}}\left[ {1 + \frac{6}{5}\varepsilon_{\text{s}} g_{0} (1 + e)} \right]^{2} + 2\varepsilon_{\text{s}}^{ 2} \rho_{\text{s}} d_{\text{s}} g_{0} (1 + e)\sqrt {\frac{{\varTheta_{\text{s}} }}{\pi }} .$$

Collisional energy dissipation:

17 $$\gamma_{{\varTheta_{\text{s}} }} = \frac{{12(1 - {\text{e}}^{2} )g_{0} }}{{d_{\text{s}} \sqrt \pi }}\varepsilon_{\text{s}}^{ 2} \rho_{\text{s}} \varTheta_{\text{s}}^{ 3 / 2} .$$

Drag coefficient:

18 $$\beta = \left\{ \begin{aligned} 150\frac{{\varepsilon_{\text{s}}^{2} \mu_{\text{g}} }}{{\varepsilon_{\text{g}} d_{\text{s}}^{ 2} }} + 1.75\frac{{\varepsilon_{\text{s}} \rho_{\text{g}} \left| {\vec{u}_{\text{g}} - \vec{u}_{\text{s}} } \right|}}{{d_{\text{s}} }}\quad \text{(}\varepsilon_{\text{g}} < 0.74\text{)} \hfill \\ 0.75\frac{{\varepsilon_{\text{s}} \varepsilon_{\text{g}} \rho_{\text{g}} \left| {\vec{u}_{\text{g}} - \vec{u}_{\text{s}} } \right|}}{{d_{\text{s}} }}C_{\text{D}} \cdot \omega (\varepsilon_{\text{g}} )\quad \text{(}\varepsilon_{\text{g}} \ge 0.74\text{)} \hfill \\ \end{aligned} \right.,$$

19 $$\omega (\varepsilon_{\text{g}} ) = \left\{ \begin{array}{*{20}l} - 0.5760 + \frac{0.0214}{{4(\varepsilon_{\text{g}} - 0.7463)^{2} + 0.0044}}\text{ (}0.74 \le \varepsilon_{\text{g}} \le 0.82\text{)} \hfill \\ - 0.0101 + \frac{0.0038}{{4(\varepsilon_{\text{g}} - 0.7789)^{2} + 0.0040}}\text{ (}0.82 < \varepsilon_{\text{g}} \le 0.97\text{)} \hfill \\ - 31.8295 + 32.8295\varepsilon_{\text{g}} \text{ (}\varepsilon_{g} > 0.97\text{)} \hfill \\ \end{array} \right.,$$

20 $$Re = \frac{{\varepsilon_{\text{g}} \rho_{\text{g}} d_{\text{s}} \left| {\vec{u}_{\text{g}} - \vec{u}_{\text{s}} } \right|}}{{\mu_{\text{g}} }},$$

21 $$C_{\text{D}} = \left\{ \begin{array}{*{20}l} \frac{{24(1 + 0.15Re^{0.687} )}}{Re}&\text{ (}Re < 1000\text{)} \hfill \\ 0.44&\text{ (}Re \ge 1000\text{)} \hfill \\ \end{array} \right..$$

Mean residence time:

22 $$\overline{t} = \frac{{\sum\limits_{i = 1}^{N} {t_{i} C_{i} (t)} }}{{\sum\limits_{i = 1}^{N} {C_{i} (t)} }}.$$

Dimensionless variance:

23 $$\sigma_{t}^{2} = \frac{{\sum\limits_{i = 1}^{N} {t_{i}^{2} C_{i} (t)} }}{{\sum\limits_{i = 1}^{N} {C_{i} (t)} }} - \overline{t}^{2} .$$

### Six-lump kinetic model

**Fig. 1:**
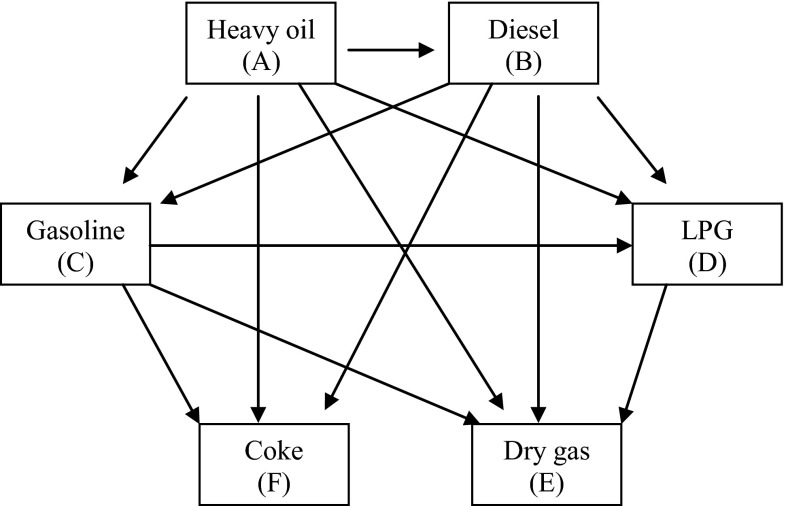
Six-lump kinetic model

Figure 1 shows the reaction network of the six-lump kinetic model, feedstock is lump A, while products were divided into five lumps, diesel (B), gasoline (C), liquefied petroleum gas (D), dry gas (E) and coke (F). The experiments were carried out on a pilot-scale FCC riser reactor. The relevant reaction parameters can be found in Table 1.

**Table 1:** The parameters of six-lump kinetic model [6]

Reaction number	*k*_0_ (s^−1^)	*E*a (KJ/mol)
A–B	601.20	59.14
A–C	2.19E+05	95.19
A–D	16.39	30.53
A–E	1.87E+03	75.58
A–F	28.49	47.10
B–C	240.46	54.20
B–D	46.08	41.07
B–E	1.56E+03	75.65
C–D	40.39	50.50
C–E	1.42	35.85
C–F	1.28	38.05
D–E	75.19	69.80

The kinetic model was established on the basis of the following assumptions [4, 6]:

1. The cracking process belongs to gas–solid phase catalytic reactions, and chemical reactions are the controlled step without considering the axial dispersion.
2. As heavy oil consists of a complex mixture of hydrocarbons, cracking is considered to be a second-order irreversible reaction, while other reactions are considered to be first-order irreversible reactions.
3. In the internal isothermal reactor, catalyst deactivation was only associated with coke content, without regarding other conditions such as poisoning inactivation.

Reaction rate constant:

24 $$K_{i} = k_{i} \times \exp ( - Ei/R/T).$$

Reaction rate equation:

25 $$R_{i} = - K_{i} \times C_{i}^{{}} \times \varphi \times \varepsilon_{\text{s}} .$$

Deactivation function:

26 $$\varphi = \frac{11.4}{{11.4 + \exp (4.29 \times C_{\text{Coke}} )}}.$$

## Problem description and boundary conditions

**Fig. 2:**
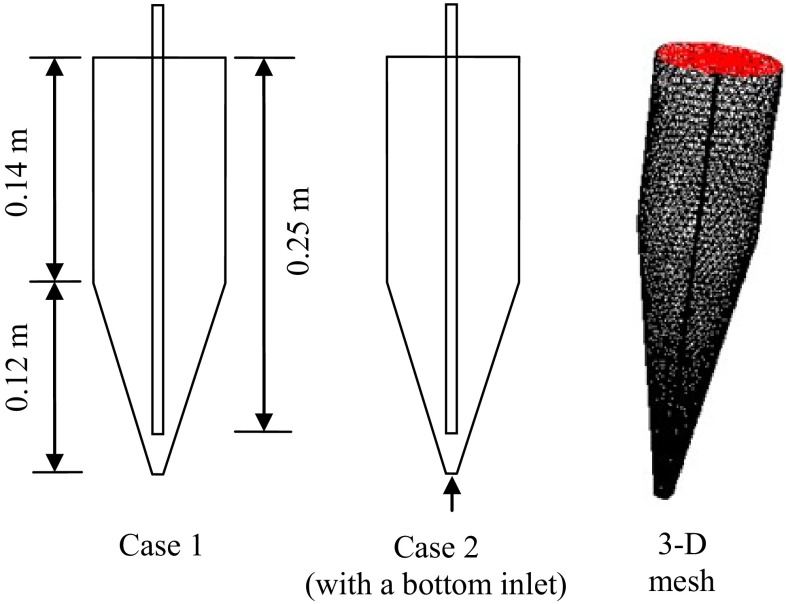
The laboratory-scale fixed fluidized bed reactor simulation diagram

GAMBIT^®^2.3 was used to compartmentalize the three-dimensional mesh region. For feeding injector, structured grids were used, while other regions were filled by unstructured grids, as shown in Fig. 2. The gas phase was considered as the primary phase, whereas the solid phase was considered as the secondary phase. The inlet was the velocity inlet, and the outlet was the pressure outlet. Case 1 shows the original reactor, while Case 2 is the modified reactor (with a bottom inlet gas, 0.01 m/s, much smaller than the feed inlet gas, 0.50 m/s). Boundary conditions used in the calculation are listed in Table 2. The experimental data were obtained from the original reactor (Case 1). The properties of the feedstock can be seen in Table 3.

**Table 2:** The simulation boundary conditions

Flow type	Laminar
Gas–solid model	Eulerian–Eulerian, with kinetic theory
Wall boundary condition	No slip
Time step used	0.0001 (s)
Restitution coefficient *e*	0.9
Max. number of iterations per time step	20
Convergence criteria	10^−3^
Maximum solid packing volume fraction	0.63
Outlet condition	Atmosphere pressure
Air density	1.225 (kg/m^3^)
Air viscosity	1.7894 × 10^−5^ (kg/m s)
Solid density	1500 (kg/m^3^)
Superficial gas velocity of feed inlet	0.50 (m/s)
Superficial gas velocity of bottom inlet (in Case 2)	0.01 (m/s)
The reaction temperature	480 (°C)

**Table 3:** The parameters of the raw material

Project	Atmospheric residue
Density (20 °C) (kg m^−3^)	909.3
Molecular weight	498.2
Kinematic viscosity (mm^2^/s)	
80 °C	37.06
Distillation (°C)	
≤350	6.23
350–500	43.94
≥500	49.83

## Results and discussion

### Effect of bottom inlet gas on the gas–solid flow

#### Gas mixing

In gas–solid fluidized bed reactors, gas mixing behavior can significantly influence the conversion and selectivity of chemical reactions. Therefore, it is important to understand the gas mixing behavior in different reactors. Gas mixing is usually studied by injecting tracer gas into experimental fluidized beds. The tracer can be injected transiently or steadily to obtain different information [16]. Transient (pulse or step change) tracer injections, often referred to as stimulus–response methods, are normally used to obtain the residence time distribution (RTD). In this work, a simulated tracer was transiently injected into the system through the feed inlet tube, and then the corresponding response at the exit tested.

**Fig. 3:**
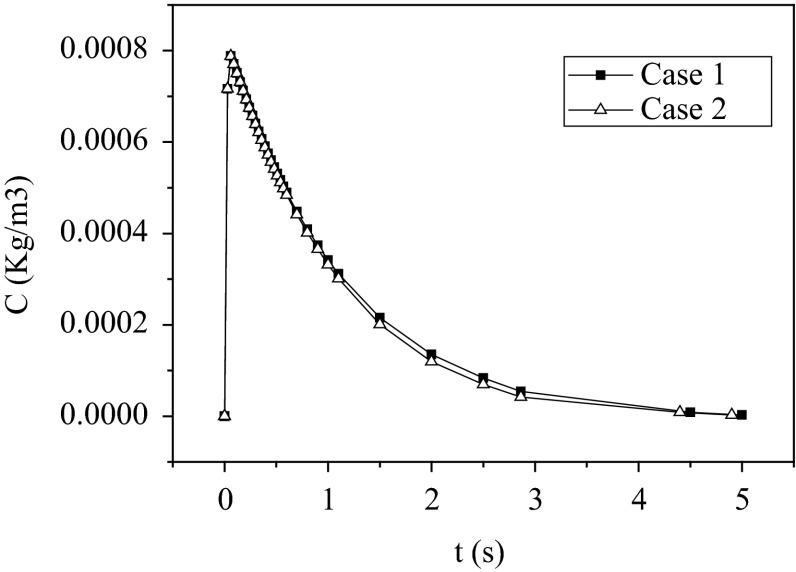
Gas residence time distribution diagram

Figure 3 and Table 4 show that with the slight bottom inlet gas, the mean residence time of the gas was shorter, and the value of dimensionless variance also decreased, which indicated that the flow pattern inside the modified reactor was closer to the plug flow. Thus, the gas back-mixing could be restrained.

**Table 4:** Mean residence time, *σ*_t_^2^ of different cases

	Bottom inlet	*t* (s)	*σ*_t_^2^
Case 1	None	0.861	0.451
Case 2	0.01 m/s	0.818	0.409

#### Catalyst distribution

**Fig. 4:**
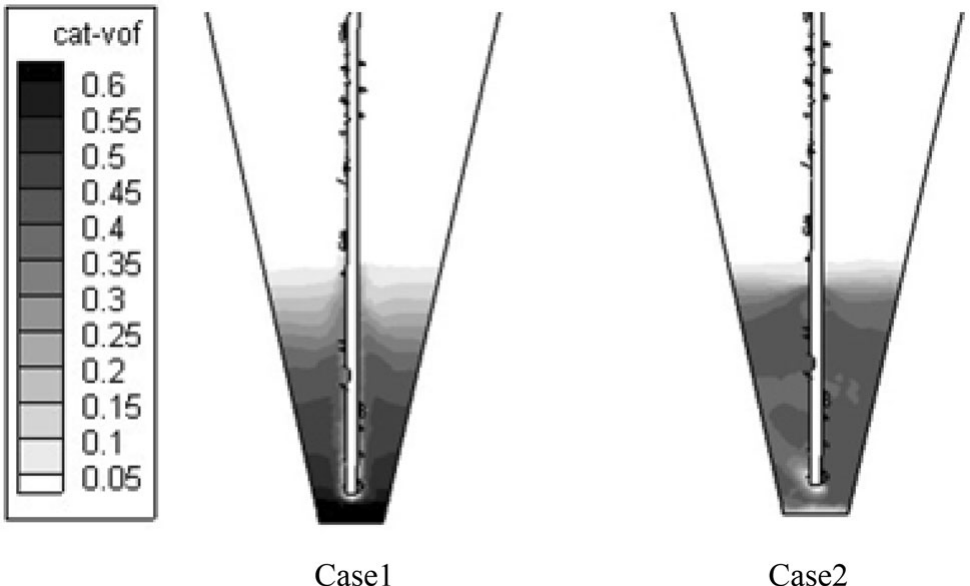
Contours of catalyst volume fraction in a vertical symmetry plane

To further understand the gas–solid flow behavior in the two reactors, the distribution of catalyst was analyzed. As seen in Fig. 4, in Case 1, the contours of catalyst volume fraction presented an uneven distribution, showing an obvious stratification, which indicated that most of the catalysts remained at the bottom of the bed and only a small part flowed up. In Case 2, the catalysts showed a more uniform distribution; the whole catalyst bed was fluidized, which could significantly improve catalyst utilization.

**Fig. 5:**
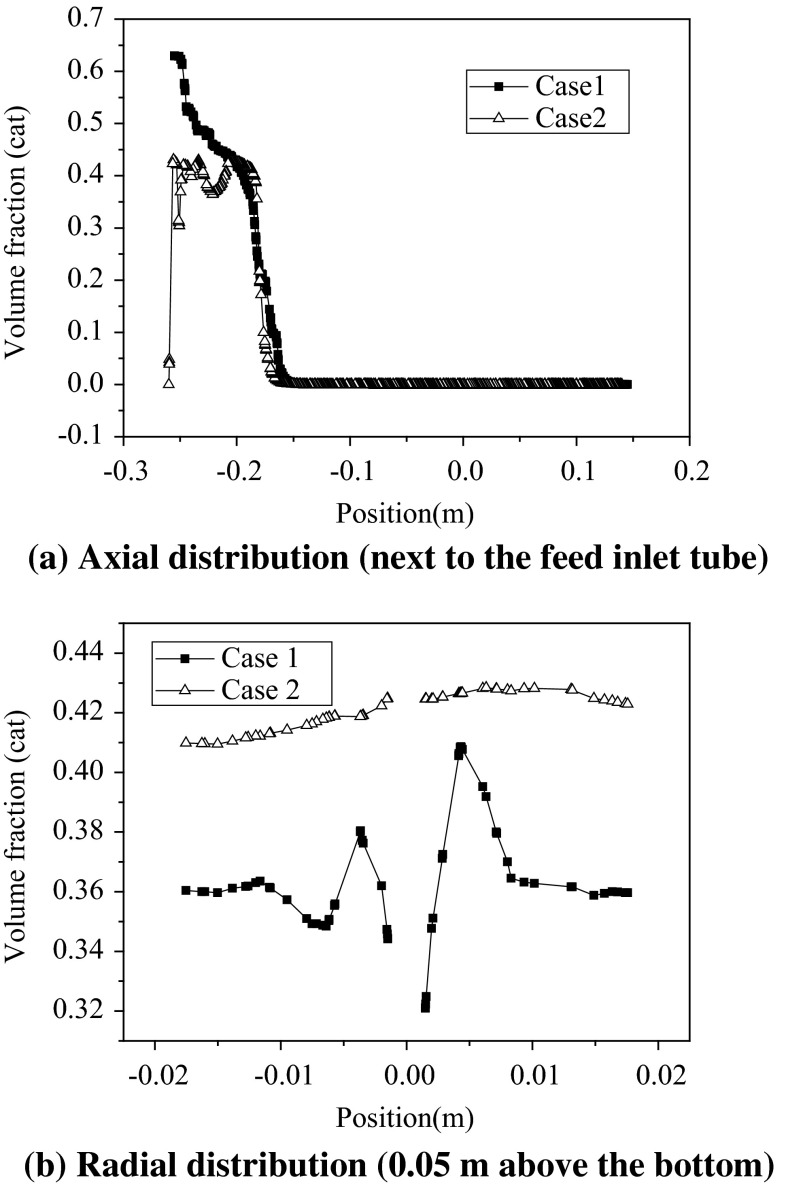
Axial and radial distributions of the catalyst

Figure 5a shows the catalyst volume fraction axial distribution along the axial height next to the feed inlet tube in the two reactors. It can be seen that the addition of the bottom inlet gas significantly improved the fluidization state of catalysts, especially in the bottom zone. Figure 5b shows the catalyst volume fraction radial distribution at a height of 0.05 m above the bottom. It can be seen that the added bottom inlet gas also obviously improved the radial distribution of the catalyst.

From Figs. 4 and 5, it can be inferred, in Case 1, that the main feed gas went through the bed along the feed injection tube; thus, the volume fractions of the catalyst near the feed injection tube were relatively low. As most of the catalysts remained at the bottom of the bed, the volume fraction of the catalyst in Case 1 was lower than that in Case 2.

For heavy oil catalytic systems, it would be better if oil gas can be fully mixed with the catalyst within the bottom mixing zone, but without back-mixing to avoid overcracking of the intermediate products after leaving the catalyst bed. Therefore, it can be inferred that the modified reactor is better for the heavy oil catalytic cracking process.

### Reaction performance

From the above analysis of gas mixing and catalyst distribution in conventional and modified fixed fluidized bed reactors, it can be found that the adding of bottom inlet gas improved the gas–solid mixing efficiency. To further understand the effect of bottom inlet gas on the cracking reactions of heavy oil, the six-lump kinetic model was incorporated into FLUENT through a UDF.

**Fig. 6:**
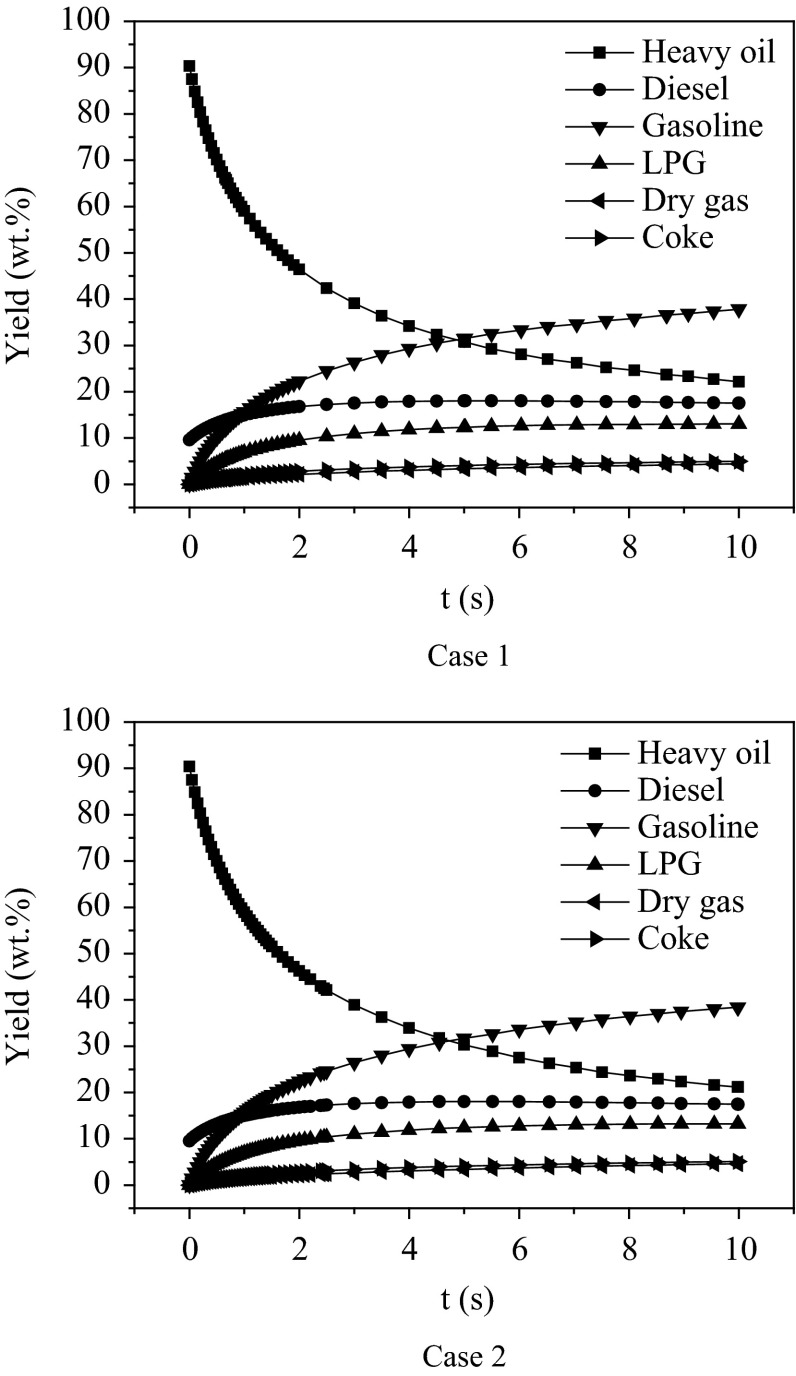
Predicted results of a six-lump kinetic model at 480 °C

It can be seen from Fig. 6 that with the increase of reaction time, the mass fraction of heavy oil first reduced sharply and then reached a plateau; the yields of gasoline, liquefied petroleum gas, dry gas and coke first increased sharply and then reached a plateau; and the mass fraction of diesel fuel increased first and then decreased. This is because at the initial time, the fresh feed is easier to be cracked, and the fresh catalyst has higher activity, leading to sharp reactions. As the reactions progress, the rest of the heavy oil components have shorter carbon chains, which are more difficult to be cracked, and the catalyst activity is reduced due to coke deposition. Thus, the reaction rate was reduced gradually.

The experimental data and the final simulated results of the established CFD model are listed in Table 5. The simulated product distribution of Case 1 is in reasonable agreement with the experimental data. The addition of the bottom inlet gas in Case 2 enhances gas–solid mixing, leading to a higher feed conversion, which increased by 1.97 wt%. With the reduction of the back-mixing degree of the generated oil gas, the modified reactor can obtain higher yields of LPG, gasoline and diesel. Even though the coke yield increased by 0.11 wt%, the selectivity of coke slightly decreased from 8.65 to 8.57 %.

**Table 5:** Predicted results and experimental data

The mass fraction (%)	Heavy oil	Diesel	Gasoline	LPG	Dry gas	Coke	Conversion
Experimental data	15.16	25.52	40.98	8.06	0.63	9.65	84.84
Simulated data of Case 1	19.15	20.54	40.83	10.04	2.45	6.99	80.85
Δ^a^	3.99	−4.98	−0.15	1.98	1.82	−2.66	−3.99
Simulated data of Case 2	17.18	21.45	41.42	10.25	2.60	7.10	82.82
Δ^b^	−1.97	0.91	0.59	0.21	0.15	0.11	1.97

^a^The simulated data of Case 1 − the experimental data

^b^The simulated data of Case 2 **−** the simulated data of Case 1

## Conclusions

1. The established gas–solid flow, heat transfer and six-lump reaction model can describe the flow state, heat transfer, mass transfer and reaction processes in fixed fluidized bed reactors. The simulated product distribution is in reasonable agreement with the experimental data.
2. The simulation results show that in the conventional fixed fluidized bed reactor, the main feed gas goes through the bed along the feed injection tube, and most of the catalysts remain at the bottom of the bed, leading to a lower gas–solid mixing efficiency.
3. The adding of slight bottom inlet gas in the modified reactor can enhance the gas–solid mixing within the lower mixing zone, and restrain gas back-mixing within the upper separating zone. Thus, the product distribution can be improved.

## List of symbols

*C*_D_: Effective drag coefficient for a particle
*d*_s_: Average diameter of particle (m)
*e*: Restitution coefficient
*g*: Gravity acceleration (m/s^2^)
*g*_0_: Radial distribution function
*S*_*c*_: Schmidt number
$$\overline{\overline{I}}$$: Unit vector
*P*: Pressure (Pa)
*Re*: Reynolds number
$\vec{u}_{\text{g}}$: Velocity of gas (m/s)
$\vec{u}_{\text{s}}$: Velocity of solid (m/s)

## Greek letters

*β*: Inter-phase momentum exchange coefficient (kg/m^3^ s)
*γ*: Dissipation of energy fluctuation (kg/m s^3^)
*ρ*: Density (kg/m^3^)
*ε*: Volume fraction
*ε*_s,max_: Maximum volume fraction of particles
*Θ*_s_: Granular temperature (m^2^/s^2^)
*τ*: Stress tensor (Pa)
*μ*_s,bulk_: Bulk viscosity (Pa s)
*μ*_s,fr_: Particle phase shear viscosity (Pa s)
*ω*: Drag coefficient correction factor
*σ*_t_^2^: Dimensionless variance
*Φ*: Internal friction angle

## Subscripts

g: Gas phase
s: Solid phase
